# Pediatric Sjögren’s Syndrome: Focus on Ocular Involvement and Diagnostic Challenges

**DOI:** 10.3390/medicina61071128

**Published:** 2025-06-23

**Authors:** Emanuela Del Giudice, Maria Carmela Saturno, Maria Grazia Fiorino, Danilo Iannetta, Luca Spadea, Vanessa Martucci, Alessia Marcellino, Mariateresa Sanseviero, Angela Mauro, Sandra Cinzia Carlesimo, Nicola Nante, Giovanni Guarducci, Leopoldo Spadea, Riccardo Lubrano, Maria Pia Paroli

**Affiliations:** 1 Pediatrics and Neonatology Unit, Department of Maternal Infantile and Urological Sciences, Santa Maria Goretti Hospital, Sapienza University of Rome, Polo Pontino, 04100 Latina, Italy; vany.mart@gmail.com (V.M.); marcellino.alessia@gmail.com (A.M.); mariateresa.sanseviero@yahoo.it (M.S.); riccardo.lubrano@uniroma1.it (R.L.); 2 Department of Sense Organs, Sapienza University of Rome, 00161 Rome, Italy; mariacarmela.saturno@virgilio.it (M.C.S.); info@mariagraziafiorino.it (M.G.F.); danilo.iannetta@uniroma1.it (D.I.); sandracinzia.carlesimo@uniroma1.it (S.C.C.); leopoldo.spadea@uniroma1.it (L.S.); 3 Post Graduate School of Public Health, University of Siena, 53100 Siena, Italy; l.spadea@student.unisi.it (L.S.); nicola.nante@unisi.it (N.N.); giovanni.guarducc@student.unisi.it (G.G.); 4 Pediatric Rheumatology Unit, Department of Childhood and Developmental Medicine, Fatebenefratelli—Sacco Hospital, 20121 Milan, Italy; angela.mauro84@gmail.com; 5 Department of Molecular and Developmental Medicine, University of Siena, 53100 Siena, Italy; 6 Healthcare Management, Local Health Authority of Ferrara, 44100 Ferrara, Italy

**Keywords:** pediatric sjögren’s syndrome, ocular involvement, tear break-up time

## Abstract

*Background and Objectives*: Pediatric Sjögren’s syndrome is a rare autoimmune disease with a heterogeneous clinical expression and limited pediatric-specific diagnostic criteria. Ocular involvement often represents an early manifestation, yet it may go unrecognized in children due to poor symptom reporting and the underuse of objective diagnostic tools. This retrospective study evaluated six pediatric patients with Sjögren’s syndrome, integrating systemic and ocular findings with a focus on early immunological and clinical markers. *Materials and Methods*: All patients underwent ophthalmological assessments, including tear break-up time, Schirmer’s test, and slit-lamp examination. *Results*: Tear break-up time values consistently indicated tear film instability (mean RE 7.4 ± 2.5 s; LE 7.7 ± 2.3 s), while Schirmer’s test showed greater variability. Slit-lamp examination revealed inhomogeneous tear films in all patients and blepharitis in 66.7%, consistent with Meibomian gland dysfunction. Systemic features included arthralgia, Raynaud’s phenomenon, fatigue, and frequent seropositivity for ANA and anti-SSA/Ro antibodies. Minor salivary gland biopsy confirmed lymphoepithelial sialadenitis in all cases. *Conclusions*: These findings highlight the importance of combining laboratory and clinical markers with ophthalmological parameters to support an early diagnosis of Sjögren’s syndrome in pediatric patients. Integrating TBUT and slit-lamp evaluation with serological and histopathological data may enhance diagnostic accuracy and guide timely, targeted intervention to prevent long-term complications.

## 1. Introduction

Sjögren syndrome (SS) was first described in 1933 by a Swedish ophthalmologist, Henrik Sjögren. SS is a chronic, systemic, and autoimmune disease characterized by lymphocytic infiltration of the exocrine glands, resulting in progressive glandular destruction, leading to mucosal dryness and sicca symptoms [1].

A wide spectrum of clinical features can occur in patients with Sjögren syndrome, ranging from exocrine involvement to extra-glandular manifestations. In pediatric patients, clinical features typically involve exocrine tissues, categorized as glandular inflammatory manifestations (such as exocrine salivary and lacrimal gland involvement or recurrent parotitis), as well as extra-glandular manifestations, particularly musculoskeletal, renal, respiratory, neurologic, cutaneous, hematologic, and gastrointestinal involvement.

Ocular signs and symptoms include surface disorders of varying severity, often with greater dryness compared to patients with non-Sjögren dry eye syndrome and healthy controls [2]. Symptoms most frequently reported by adults with primary SS include foreign body sensation, altered tearing (either reduced or increased), itching, and blurred vision. The slit lamp examination shows signs of dry eye as redness, conjunctival keratinization with chalasis, and punctate or filamentous keratitis [3]. In some cases, anterior blepharitis and meibomian gland dysfunctions can be found [4].

Sjögren syndrome is usually prevalent in middle-aged women [3], while pediatric cases are very rare. Diagnosis in children is particularly challenging, given the broad spectrum of clinical manifestations and the difficulty that young patients often have in communicating their symptoms [5,6].

The rarity of the condition, combined with its heterogeneous clinical presentation and lack of pediatric-specific diagnostic criteria, often results in delayed diagnosis and under-recognition of ocular involvement [5,6].

Moreover, there are no gold standard diagnostic criteria for Pediatric Sjögren’s syndrome (pSS), although the modified ACR/EULAR 2016 adult criteria were used frequently [7]. Thus, diagnosis relies heavily on clinical expertise based on a combination of patient history, clinical examination, functional tests of the exocrine glands, and serological or histological evidence.

However, a considerable proportion of pediatric SS patients present predominantly with extra-glandular manifestations without sicca symptoms. This contrasts with adult SS patients, in whom sicca symptoms are present in 80–100% of cases and often constitute the predominant presenting feature [6].

Consequently, diagnostic criteria designed for adults often fail to identify many children with SS, leading to underdiagnosis and delayed access to appropriate care [6,8].

An accurate and detailed medical history is, therefore, crucial for the correct management and therapy of patients with pSS. The prevalence of ocular symptoms in pediatric SS cases ranges from 35% to 70% [9]. Even though classical dry eye symptoms (xerophthalmia or keratoconjunctivitis sicca) may not always be clearly expressed by children, a history of red eyes, foreign body sensation, pain, photosensitivity, and itching should raise suspicion [8]. Therefore, an ophthalmological examination, including Schirmer’s test and evaluation of subclinical keratoconjunctivitis sicca using Rose bengal or fluorescence stains, such as tear break-up time (TBUT), is recommended [8].

Despite these recommendations, ocular involvement remains frequently underdiagnosed in pediatric SS, particularly when symptoms are mild or attributed to other causes (e.g., allergies, screen use, environmental dryness) [5,6]. TBUT, as a non-invasive and sensitive test for tear film instability, may offer an early diagnostic clue even in the absence of overt dryness complaints [8].

Moreover, early identification of ocular signs can serve as a gateway to systemic diagnosis, particularly in cases where rheumatologic symptoms are subtle or evolving.

The aim of this study was to identify clinical systemic features and ocular manifestations through early altered ophthalmological tests in patients diagnosed with pSS.

By focusing on tear film abnormalities, including TBUT and Schirmer’s test, and correlating them with systemic and serological findings, our study seeks to support the role of ophthalmologic evaluation as a key component in the diagnostic and management pathway of pediatric SS.

## 2. Materials and Methods

### 2.1. Patients

We conducted a retrospective analysis of all consecutive pediatric patients with a diagnosis of primary or secondary pSS, with the disease onset before the age of 18 years referred to the Ocular Immunology Service, Department of Sense Organs, Policlinico Umberto I, Sapienza University of Rome, and Department of Pediatrics Santa Maria Goretti Hospital, Sapienza University of Rome from January 2013 to January 2023.

Patients were eligible for inclusion if they were diagnosed with pSS by pediatric rheumatologists according to the criteria proposed by Bartůnková et al. [10]. Exclusion criteria included a history of malignancy, other systemic comorbidities, chronic metabolic or infectious diseases, and prior treatment with drugs potentially affecting ocular function.

For each patient, informed written consent was obtained from parents or caregivers, authorizing the anonymous use of clinical data for research purposes. The study protocol was approved by the local ethics committee and conducted in accordance with the Declaration of Helsinki of 1975 [11].

### 2.2. Data Collection

During the ophthalmological and pediatric evaluation, demographic data, laboratory parameters, and disease-related variables were collected, including sex, age at pSS diagnosis, and disease duration at the time of the ophthalmological examination. Each patient completed a symptom questionnaire [12] about their symptoms, and a complete eye examination was then performed on all patients. The diagnosis and classification of keratoconjunctivitis sicca were based on the detection of qualitative and quantitative alterations of the tear film and the evidence of ocular surface damage, assessed by slit-lamp examination and specific tests, including tear break-up time (TBUT) and Schirmer’s test, according to the Sjögren’s International Collaborative Clinical Alliance (SICCA) criteria [13].

Schirmer’s test I (without anesthesia) was performed by placing standardized Whatman filter paper strips in the lateral third of the lower eyelid fornix of both eyes. Patients were instructed to keep their eyes closed during the 5 min testing period. The length of wetting, measured in millimeters from the fold, was recorded. A value of ≤5 mm after 5 min was considered indicative of severe aqueous tear deficiency [8].

TBUT was evaluated after the instillation of a drop of fluorescein into the inferior conjunctival fornix. The patient was instructed to blink several times to ensure even distribution of the dye and then to keep their eyes open without blinking. Using slit-lamp biomicroscopy with a cobalt blue filter, the interval between the last complete blink and the appearance of the first dry spot on the corneal surface was measured three times, and the average value was recorded. A TBUT of ≤10 s was considered abnormal [8].

Both tests were conducted under controlled ambient conditions (temperature and humidity) and in the same time window (late morning) to reduce environmental influences and diurnal variations. All procedures were carried out by the same trained ophthalmologist, ensuring consistency in technique and interpretation.

Moreover, systemic activity was evaluated by the European League Against Rheumatism (EULAR) Sjögren’s Syndrome Disease Activity Index (ESSDAI) according to EULAR recommendations [14]. Minor salivary gland biopsy confirmed lymphoepithelial sialadenitis in all cases [15].

Laboratory parameters were analyzed, including erythrocyte sedimentation rate (ESR), C-reactive protein (CRP), antinuclear antibodies (ANA), rheumatoid factor (RF), and anti-SSA/Ro and anti-SSB/La antibodies. When available, data from salivary gland ultrasonography were also collected.

### 2.3. Statistical Analysis

All data were summarized and displayed as mean ± SD for the continuous variables. Categorical data were expressed as frequencies and percentages. The chi-square test was used to compare categorical variables. Statistical significance was set at *p*-value < 0.05. SPSS version 21 was used to perform all statistical evaluations (SPSS Inc., Chicago, IL, USA).

## 3. Results

A total of six patients (four females, 66.7%) were included in the cohort, all diagnosed with pSS, fulfilling the criteria proposed by Bartůnková et al. [10]. Three patients had a diagnosis of primary Sjögren’s syndrome, while the remaining three had secondary Sjögren’s syndrome associated with undifferentiated connective tissue disease.

Baseline characteristics are summarized in Table 1.

The median age at first ophthalmological evaluation was 15.8 years (range: 12–21 years), while the median age at disease onset was 9.5 ± 2.8 years, and the mean age at diagnosis of primary Sjögren’s syndrome (pSS) was 11.0 ± 2.7 years. The most common clinical manifestations at diagnosis included arthralgia, sicca symptoms, xerostomia in 33.3% and xerophthalmia in 83.3% of patients, Raynaud phenomenon (66.7%), and fatigue (66.7%).

Regarding immunological findings, 50% of patients tested positive for antinuclear antibodies (ANA, titer 1:160), and 50% were positive for anti-SSA/Ro antibodies. All patients underwent salivary gland ultrasonography, which showed abnormalities in five out of six cases (83.3%), and all underwent minor salivary gland biopsy, which revealed lymphoepithelial sialadenitis.

Following the administration of the diagnostic questionnaire, all patients reported symptoms of ocular discomfort, specifically ocular burning and itching sensation, that worsened in the evening and after prolonged visual tasks such as reading at school.

On slit-lamp examination, all patients (100%) exhibited an irregular tear film, and four (66.7%) showed signs of anterior blepharitis, including erythema, thickening of the eyelid margins, lash debris, and collarettes at the base of the eyelashes.

Additional findings included conjunctival hyperemia and mild punctate epitheliopathy of the interpalpebral area in four patients (66.7%), consistent with tear film instability and ocular surface involvement. Lid margin inflammation and debris were also observed in patients with blepharitis, further supporting the multifactorial nature of dry eye disease in this cohort.

Moreover, ocular surface function tests showed mean Schirmer I test values: right eye (RE) 13 mm ± 4.6, left eye (LE) 12.5 mm ± 3.7, and a mean tear break-up time (TBUT): RE 7.4 s ± 2.5; LE 7.7 s ± 2.3.

## 4. Discussion

The diagnosis of primary Sjögren’s syndrome (pSS) in pediatric patients is particularly challenging due to the difficulty young children face in clearly communicating their symptoms and the lack of a standardized diagnostic tool for this age group [6,8].

Our study highlights the importance of comprehensive ophthalmologic assessment, particularly emphasizing tear film break-up time (TBUT) as a sensitive, non-invasive indicator of tear film instability, more reliable than Schirmer’s test in this cohort.

While Schirmer’s test evaluates aqueous tear production, it does not assess Meibomian gland function or the lipid layer integrity, both critical for tear film stability. Autoimmune-mediated meibomian gland dysfunction (MGD) in pSS contributes to evaporative dry eye through ductal obstruction and lipid layer deficiency. In our cohort, TBUT values were consistently abnormal, with a mean of 7.4 ± 2.5 s in the right eye and 7.7 ± 2.3 s in the left eye, supporting significant tear film instability. Conversely, Schirmer’s test results were highly variable and less informative.

All patients exhibited irregular tear films on slit-lamp examination, and 66.7% showed signs of blepharitis, correlating with MGD presence. These findings align with previous reports, indicating frequent Meibomian gland involvement in pSS, even in pediatric populations [3,14,15,16,17]. Previous studies demonstrated reduced gland expressibility, increased dropout, and inflammatory infiltration around Meibomian glands in pSS [18,19,20,21], attributed to local cytokine-mediated damage and androgen deficiency [3].

It is particularly noteworthy that even in a small cohort, TBUT proved to be a consistent marker of tear film instability, reinforcing its value in the early detection of ocular surface dysfunction. Given the young age of the patients and the subtlety of symptoms, a sensitive, repeatable, and well-tolerated test such as TBUT should be considered a cornerstone of ophthalmologic evaluation in suspected pediatric pSS.

Importantly, normative values for TBUT and Schirmer’s test in children are lacking [9], complicating the interpretation of tear tests. However, TBUT remains more sensitive in detecting tear film instability, particularly in evaporative dry eye, which may occur even with preserved aqueous secretion. Therefore, a normal Schirmer test does not exclude significant ocular surface disease in Sjögren’s syndrome, where rapid evaporation due to lipid layer dysfunction predominates [21,22,23,24,25,26,27].

Thus, for a robust ophthalmologic evaluation in pediatric pSS, TBUT and direct assessment of Meibomian gland function should complement Schirmer’s test.

Moreover, behavioral factors, such as reduced blinking during screen time, can exacerbate ocular surface disease in children. If unrecognized, dry eye may progress to corneal melting, perforation, or infection, risking permanent vision loss. Early diagnosis and symptomatic management are essential to prevent serious complications.

In pediatric practice, ocular symptoms such as burning, itching, and visual fatigue may easily be overlooked or misattributed to other causes, especially when systemic signs are predominant. This emphasizes the need for routine, targeted ophthalmologic evaluation in rheumatologic settings, even in the absence of classic ”sicca” complaints.

Systemically, our cohort showed frequent arthralgia (83.3%), Raynaud’s phenomenon (66.7%), and fatigue (66.7%). Laboratory results included 50% ANA positivity, 50% anti-SSA/Ro positivity, and polyclonal hypergammaglobulinemia in 66.7%. Salivary gland ultrasound abnormalities were identified in 66.7% of patients, and all underwent confirmatory minor salivary gland biopsy showing lymphoepithelial sialadenitis, consistent with previous reports of biopsy sensitivity (63.9–85.7%) and specificity (61.2–100%) [3].

Also, the presence of xerostomia is a significant contributor to morbidity, impacting dental health through both hyposalivation and altered oral microbiota [28]. Furthermore, pSS may involve multiple exocrine glands, causing respiratory, hepatic, pancreatic, and gynecologic complications. Extra-glandular manifestations, including arthritis, interstitial lung disease, and renal and neurological involvements, may coexist [3].

Despite the critical role of ophthalmologic findings in ACR/EULAR classification, ophthalmologic tests are underutilized in pediatric practice [29,30,31]. Although several serum biomarkers (anti-Ro/SSA, anti-La/SSB, ANA, anti-M3R antibodies, RF) have been proposed [18,31,32], none is sufficient for a definitive diagnosis.

Even though minor salivary gland biopsy remains the gold standard for definitive diagnosis, the combination of subjective symptoms, abnormal TBUT, and slit-lamp findings strongly supports the need for routine ophthalmologic screening in pediatric patients with suspected or confirmed pSS, even in the absence of overt sicca symptoms.

Salivary gland ultrasonography (SGUS) is a safe, non-invasive tool increasingly recognized for its role in diagnosing pediatric primary Sjögren’s syndrome. It can detect characteristic glandular abnormalities even when sicca symptoms are minimal or absent, facilitating earlier diagnosis. Recent pediatric studies have demonstrated SGUS’s good sensitivity and specificity, supporting its integration into the routine evaluation to improve timely identification and management of glandular involvement in children with pSS [33].

Integrating ophthalmologic evaluation into the diagnostic workflow for pediatric pSS may facilitate earlier diagnosis, guide treatment decisions, and help monitor response to therapy over time. As ocular involvement can precede systemic manifestations, it may also serve as an early disease indicator.

Thus, early ophthalmologic screening, integrating subjective symptoms, TBUT, and slit-lamp findings, remains crucial for timely diagnosis. Prompt initiation of artificial tears, eyelid hygiene, and anti-inflammatory therapies can significantly improve quality of life and prevent long-term complications [34].

This study has several limitations. The sample size was relatively small, limiting the generalizability of the findings. Given the rarity of primary Sjögren’s syndrome (pSS) in pediatric populations, larger multicenter studies are needed to validate these observations. Moreover, the study design was cross-sectional; thus, we could not assess the progression of ocular or systemic manifestations over time. Additionally, reference values for TBUT and Schirmer’s test in children are lacking, which may affect the interpretation of results and highlight the need for pediatric-specific reference standards. Finally, the subjective reporting for some symptoms in a pediatric population may have introduced bias, given the inherent difficulties young children may have in accurately describing ocular or oral dryness. Future research should focus on developing pediatric-specific diagnostic criteria and non-invasive biomarkers to enhance early detection.

Despite these limitations, our findings support the integration of targeted ophthalmologic evaluation in the multidisciplinary management of pediatric pSS. Future research should focus on developing validated pediatric-specific diagnostic criteria, standardizing ocular surface testing protocols, and identifying non-invasive biomarkers to enhance early detection and monitoring of disease activity.

## 5. Conclusions

In conclusion, close collaboration between pediatric rheumatologists and ophthalmologists is essential for the early identification of ocular involvement in pediatric primary Sjögren’s syndrome (pSS), even in the absence of overt sicca symptoms. This is particularly important in children, where symptoms may be nonspecific or underreported and where early ocular signs may represent the first clinical manifestation of the disease.

A multidisciplinary approach is critical to improving patient outcomes, preventing severe ocular and systemic complications, and enhancing the overall quality of life in affected children. The integration of systematic ophthalmologic screening, such as tear break-up time (TBUT) measurement and slit-lamp examination, into the routine assessment of children with suspected or confirmed pSS can support timely diagnosis and the prompt initiation of appropriate local therapies.

In clinical practice, a structured diagnostic algorithm that combines subjective symptom questionnaires with objective, non-invasive tests could serve as a feasible model for the early detection of ocular surface disease in this population.

Further longitudinal, multicenter studies are warranted to establish pediatric-specific diagnostic thresholds, better understand the long-term progression of ocular involvement in pSS, and evaluate the impact of early ophthalmologic intervention on both visual prognosis and systemic disease outcomes.

## Figures and Tables

**Table 1:** Baseline Characteristics of Participants.

Demographic Characteristic	
Female, *n* (%)	4 (66.7%)
Age, (range) years	15.8 (12; 21)
Age at diagnosis (mean ± SD) years	11.0 ± 2.7
Age at disease onset (mean ± SD) years	9.5 ± 2.8
ESSDAI (mean ± SD)	4.0 ± 2.5
**Laboratory**	
ANA positive, *n* (%)	3 (50%)
RF+, *n* (%)	-
ESR [mm/h], positive *n* (%)	3 (50%)
CRP [mg/dL]), positive *n* (%)	1 (16.7%)
Platelets [cell ×1.000/mm^3^] mean ± SD	250.1 ± 71.8
Hemoglobin [g/dL] mean ± SD	12.35 ± 1.48
Hypertransaminasemia, *n* (%)	-
Hyperamylasemia, *n* (%)	1 (16.5%)
Leukopenia, *n* (%)	-
Low C3 levels, *n* (%)	2 (33.3%)
Low C4 levels, *n* (%)	1 (16.7%)
Polyclonal Hypergammaglobulinemia, *n* (%)	4 (66.7%)
Anti ds DNA, positive *n* (%)	-
ENA, positive *n* (%)	3 (50%)
SSA/Ro	3 (50%)
SSB/La	-
**Clinical manifestations**	
Xerostomy, *n* (%)	2 (33.3%)
Xerophthalmia, *n* (%)	5 (83.3%)
Photosensitivity, *n* (%)	-
Dental caries, *n* (%)	-
Parotitis, *n* (%)	-
Lymphadenopathy, *n* (%)	2 (33.3%)
Telogen effluvium, *n* (%)	-
Arthralgia, *n* (%)	5 (83.3%)
Arthritis, *n* (%)	3 (50%)
Myalgia, *n* (%)	2 (33.3%)
Fever, *n* (%)	-
Raynaud’s phenomenon, *n* (%)	4 (66.7%)
Fatigue, *n* (%)	4 (66.7%)
Rash, *n* (%)	-
**Documentation of parotid gland involvement**	
Salivary gland abnormalities in ultrasound, *n* (%)	5 (83.3%)
Sialography	-
**Ophthalmological tests**	
At SLE: irregular tear film	6 (100%)
At SLE: signs of anterior blepharitis	4 (66.7%)
Schirmer’s test (mean ± SD)	RE 13 mm ± 4.6; LE 12.5 mm ± 3.7
TBUT (mean ± SD)	RE 7.4 s ± 2.5; LE 7.7 s ± 2.3

ANA, antinuclear antibody; Anti ds DNA, Anti-double stranded DNA; Extractable nuclear antigen (ENA); c-reactive protein (CRP), erythrocyte sedimentation rate (ESR); ESSDAI, EULAR Sjögren’s Syndrome Disease Activity Index; Rheumatoid factor (RF), Slit lamp exam (SLE).

## Data Availability

All relevant data are reported in this article. Additional details can be provided by the corresponding author upon reasonable request.

## Abbreviations

The following abbreviations are used in this manuscript:

ANA	antinuclear antibodies
ACR	American college rheumatology
CRP	C-reactive protein
ESR	erythrocyte sedimentation rate
EULAR	European alliance of associations for rheumatology
MGD	Meibomian gland dysfunction
RF	rheumatoid factor
SICCA	Sjögren’s International Collaborative Clinical Alliance
pSS	pediatric Sjögren’s syndrome
TBUT	tear break-up time
